# Assessing the population’s correct knowledge of malaria in Malaysia: a vital component for malaria elimination certification

**DOI:** 10.1186/s12936-023-04704-1

**Published:** 2023-09-12

**Authors:** Faizul Akmal Abdul Rahim, Mohd Farihan Md Yatim, Mohd Hatta Abdul Mutalip, Mohd Amierul Fikri Mahmud

**Affiliations:** Centre for Communicable Diseases Research, Institute for Public Health, National Institutes of Health, Ministry of Health, Shah Alam, Malaysia

**Keywords:** Malaria, Malaria elimination, Knowledge, Population-based survey, Malaysia

## Abstract

**Background:**

Malaria remains a public health problem in Malaysia despite a decline in the number of cases in recent years. Public knowledge of malaria is essential to achieving and maintaining malaria elimination. Therefore, this survey assessed the prevalence of people who had ever heard of malaria, had correct knowledge of malaria transmission, symptoms, risk activities, and prevention measures in the Malaysian population, and identified the associated factors involved.

**Methods:**

The data were obtained from the National Health and Morbidity Survey (NHMS) which was conducted from September to October 2020. A cross-sectional survey with five structured questionnaires using the method of computer-assisted telephone interviews (CATI) was used to collect data. The socio-demographic characteristics such as age, gender, ethnicity, nationality, marital status, educational level, and occupation were recorded. Data were analysed using STATA SE Version 16. Associations between variables were tested using chi-square and logistic regression, with the level of statistical significance set at p < 0.05.

**Results:**

Out of 3085 respondents who participated in this survey, 2491 had ever heard of malaria with a prevalence of 76.1% (95% CI 70.5, 80.9). The prevalence of correct knowledge of malaria transmission was 53.9% (95% CI 50.2, 57.7), symptoms 24.1% (95% CI 21.2, 27.2), risk activities 34.0% (95% CI 29.3, 39.1) and prevention measures 59.7% (95% CI 55.5, 63.7). Multivariate analysis showed that age, ethnicity, and educational level were significantly associated with correct malaria transmission and risk activities. In addition, age and educational level were significantly associated with correct malaria symptoms. Subsequently, only the variable ethnicity showed a significant association with the correct malaria prevention measures.

**Conclusion:**

In general, the prevalence of ever heard of malaria was considered high since the survey was performed based on a non-specific malaria population. However, the prevalence of correct knowledge of malaria symptoms and risk activities were considered low. It is concluded that educational level is one of the major factors associated with correct malaria knowledge, along with age and ethnicity. Therefore, based on these findings, targeted intervention and innovation should be planned by malaria programme managers to sustain malaria elimination in Malaysia.

**Supplementary Information:**

The online version contains supplementary material available at 10.1186/s12936-023-04704-1.

## Background

Malaria is a tropical disease caused by *Plasmodium* parasites and is considered one of the most important infectious diseases worldwide. In 2019, there were estimated 229 million cases of malaria in 87 malaria-endemic countries, an increasing trend compared to 218 million cases of malaria in 2015 [1, 2]. Nevertheless, in the Southeast Asia region, malaria cases were reduced by 73%, from 23 million in 2000 to 6.3 million in 2019. In addition, malaria incidences in this region were reduced by 78%, from 18 cases per 1000 population at-risk in 2000 to 4 cases in 2019. However, the malaria incidence in the South-East Asia region still contributed about 3% to the global burden of malaria cases. [1].

In Malaysia, the Malaria Eradication Programme was established in 1967. Since then, the number of malaria cases has decreased significantly. A tremendous drop in the number of malaria cases was reported from 243,870 cases in 1961 to 44,226 cases in 1980 [3]. The programme was then further strengthened and improved, with only 2302 malaria cases reported in 2016 [4]. Currently, Malaysia has managed to maintain zero cases of indigenous human malaria infection status since 2018 and qualifies for the World Health Organization (WHO) Human Malaria Elimination Certification in 2021 [1].

Nevertheless, sustaining malaria elimination requires multiple actions and integrated strategies. Public knowledge regarding malaria is considered an important assessment component of malaria elimination certification. According to the WHO, good knowledge of malaria causes, signs and symptoms, mode of transmission, and prevention measures will encourage the implementation of malaria prevention strategies, improve health-seeking behaviour, and significantly increase the sustainability of malaria elimination programmes [5, 6]. Furthermore, only a minimum level of education is needed to develop a correct understanding of malaria transmission, thus, attitude adjustment toward malaria control and elimination can be made [7–9]. Additionally, knowledge of malaria prevention methods will likely influence household practices in controlling the disease. Therefore, it is important to establish community knowledge and practices for malaria control, especially in rural areas which endure a high disease burden [10, 11].

In the past decade, several surveys have been conducted in malaria-risk areas in Malaysia to assess communities' knowledge of malaria [12–14]. However, there are still not enough studies assessing population knowledge, which is more emphasized. Therefore, this population-based study was conducted to provide baseline knowledge of malaria among Malaysians. These findings could be utilized by the Ministry of Health (MOH) to strengthen current strategies for achieving malaria elimination certification. In parallel with the implementation of malaria elimination in Malaysia, this survey was conducted to assess the prevalence of people who had ever heard of malaria, correct knowledge of malaria transmission, symptoms, risk activities, and prevention measures in the Malaysian population and identify the associated factors involved (Additional file 1: Tables S1 and S2).

## Methods

### Sampling design

The National Health and Morbidity Survey (NHMS) is a cross-sectional survey with a complex survey design. The survey was conducted from September to October 2020. The samples represent the entire population aged 15 and above living in non-institutional housing units in Malaysia, regardless of citizenship. The survey excluded people living in institutional living quarters, such as hotels, hostels, hospitals, prisons, boarding houses, and nursing homes. This survey used a two-stage stratified random sampling technique to ensure national representativeness. The primary stratum consists of all the states of Malaysia including the Federal Territories and the secondary stratum consists of urban and rural strata within the primary stratum. The sampling procedure consisted of two stages, with the primary sampling unit being the enumeration blocks and the secondary sampling unit being the living quarters within each sampled enumeration block. Details of the sampling methodology and sampling weights applied for the national representativeness are explained in the NHMS 2020 communicable disease report [15].

### Sample size determination

The sample size was calculated using a single proportion formula for the estimation of prevalence.

$$n_{SRS} \ge \frac{{Z_{{{\alpha \mathord{\left/ {\vphantom {\alpha 2}} \right. \kern-0pt} 2}}}^{2} P\left( {1 - P} \right)}}{{e^{2} }}$$

The sample size calculation was based on a few criteria as below:

- Variance of the proportion of the variable of interest, for this study the lowest prevalence for the malaria scope which was 14% on the awareness of malaria was selected [16],
- Margin of error (e) of 0.05,
- Confidence interval of 95%

A few adjustments were also made to ensure optimum sample size:

- Adjusted for the finite population (Based on the 2020 projected Malaysian population)

$$n \ge \frac{{n_{SRS} }}{{1 + \frac{{n_{SRS} }}{N}}}$$

b. Adjusted for the design effect (deff) of 2.0, where n(complex) = n(srs)*deff.

c. Adjusted the n(complex) taking into account expected non-response rates of 35% for interviews conducted via CATI, n(adj) = n(complex)* (1 + non-response rate).

Hence, the optimum sample size required was 370 respondents. After adjustment with the 35% non-response rates, a total of 569 respondents were required for a national prevalence estimate. Further sample size adjustment was made according to the need of the analysis, whether the prevalence estimate was at national, urban, or rural levels. After adjustment, a total of 288 living quarters was selected from 113 of the total enumeration blocks in Malaysia with a factor of 2.5 eligible respondents aged 15 years old per living quarter yielded an estimated 720 respondents required for this malaria study.

### Questionnaire survey

Five structured questionnaires adapted from the Malaria Indicator Survey were used for data collection [17]. A validated bilingual (Bahasa Melayu and English) and pre-tested questionnaires were used. The questionnaire manual served as a guide for data collection. Respondents who answered “yes” to the first question moved on to the second through fifth questions. Respondents who answer “no” to the first question will end this survey. Respondents must answer “yes” or “no” to all answer options from the second through fifth questions. Respondents who answered all answer options correctly are declared as having the correct knowledge. All related questions are described below:

1. Respondents had ever heard of malaria.
2. Knowledge about malaria transmission: (1) Through mosquito bites, (2) Through food/water, (3) Through body contact, (4) Through the air.
3. Knowledge about malaria symptoms: (1) Fever, chills, and rigors, (2) Flu and rashes, (3) Prolonged cough and constipation.
4. Knowledge about malaria risk activities: (1) Fishing in the swamp or forest, (2) Recreational activities in the forest, (3) Collecting agricultural produce, (4) Eating contaminated food, (5) Inhaling polluted water.
5. Knowledge about malaria preventive measures: (1) Taking an antimalarial medication or applying mosquito repellent during activities in the forest area, (2) wearing protective clothing during outdoor activities in the farm/forest area, (3) Sleeping under insecticide-treated nets, (4) spraying insecticide on the wall surface.

### Data collection

Since this survey was conducted during the COVID-19 pandemic, interviews were done through computer-assisted telephone interviews (CATI) instead of face-to-face meetings with respondents. The interviews were conducted by ten trained research assistants who were fluent in Bahasa Melayu, English, and other Malaysian dialects. Unsuccessful CATI surveys were attributed to respondents who hung up the phone, did not answer calls, had incorrect phone numbers, a phone number that was no longer in service, refused to participate, or faced a language barrier.

### Data analysis

The data analysis was performed on account of the complex sample design with STATA SE Version 16 (Stata Corp, College Station, TX USA). Basic sociodemographic characteristics were shown as percentages and frequencies, with 95% confidence intervals, along with weighted estimates. Analyses were stratified by area of residence (urban/rural) due to the influence of neighbourhood status on health knowledge, self-efficacy, and practice. Chi-square tests were used to assess significant differences in correct knowledge about transmission, symptoms, risk activities, and prevention measures between urban and rural areas. Logistic regression was used to examine the factors associated with correct knowledge about malaria in Malaysia. The significance level was set at p < 0.05.

## Results

### Socio-demographic characteristics of respondents

A total of 3,085 respondents aged 15 and older were included in the survey. The basic socio-demographic characteristics of the respondents were presented as frequencies and percentages (Table 1). The mean age was 39.68 (SD ± 16.03). Most of the respondents were 30–39 years old (21.2%), female (54.0%), a prominent ethnicity was Malay (63.9%) and the majority of respondents were Malaysian citizens (93.5%). In addition, most respondents were married/living with a partner (64.5%), had secondary education (49.8%), and worked as private employees (28.5%). There was a significant association among respondents who participated in this survey by location (urban/rural) with variables such as ethnicity (P = 0.001) and educational level (P = 0.008).

**Table 1:** Sociodemographic characteristics of the respondents (n = 3085)

Variable	Population			P-value
	Urban n = 1718 (%)	Rural n = 1367 (%)	Total n = 3085 (%)	
Age (39.68 ± 16.03)				0.325
15–19	187 (10.9)	154 (11.3)	341 (11.1)	
20–29	365 (21.3)	282 (20.6)	647 (21.0)	
30–39	404 (23.5)	250 (18.3)	654 (21.2)	
40–49	286 (16.7)	259 (18.9)	545 (17.7)	
50–59	245 (14.3)	229 (16.8)	474 (15.4)	
60–69	172 (10.0)	144 (10.5)	316 (10.2)	
70 & above	59 (3.4)	49 (3.6)	108 (3.5)	
Sex				0.630
Male	790 (46.0)	629 (46.0)	1419 (46.0)	
Female	928 (54.0)	738 (54.0)	1666 (54.0)	
Ethnicity				0.001*
Malay	1030 (60.0)	942 (68.9)	1972 (63.9)	
Chinese	233 (13.6)	34 (2.5)	267 (8.7)	
Indian	106 (6.2)	33 (2.4)	139 (4.5)	
Other Bumiputras	266 (15.5)	273 (20.0)	539 (17.5)	
Others	83 (4.8)	85 (6.2)	168 (5.5)	
Citizenship				0.490
Malaysian citizen	1617 (94.1)	1268 (92.8)	2885 (93.5)	
Non-Malaysia citizen	101 (5.9)	99 (7.2)	200 (6.5)	
Marital Status				0.703
Single	512 (29.8)	375 (27.4)	887 (28.8)	
Married/Living with a partner	1085 (63.2)	906 (66.3)	1991 (64.5)	
Widowed (er)/divorcee	121 (7.0)	86 (6.3)	207 (6.7)	
Education Level				0.008*
No formal education	50 (2.9)	64 (4.7)	114 (3.7)	
Primary education	245 (14.3)	318 (23.3)	563 (18.3)	
Secondary education	806 (46.9)	731 (53.5)	1537 (49.8)	
Tertiary education	617 (35.9)	254 (18.6)	871 (28.2)	
Occupation				0.059
Government employee	187 (10.9)	111 (8.1)	298 (9.7)	
Private employee	530 (30.9)	348 (25.5)	878 (28.5)	
Self-employed	227 (13.2)	283 (20.7)	510 (16.5)	
Unpaid worker/Homemaker/caregiver	301 (17.5)	301 (22.0)	602 (19.5)	
Student	195 (11.4)	136 (10.0)	331 (10.7)	
Not working (unemployed, health problem, old age, retiree)	272 (15.8)	184 (13.5)	456 (14.8)	
Others	6 (0.4)	4 (0.3)	10 (0.3)	

### Response from respondents who had ever heard of malaria

**Fig. 1:**
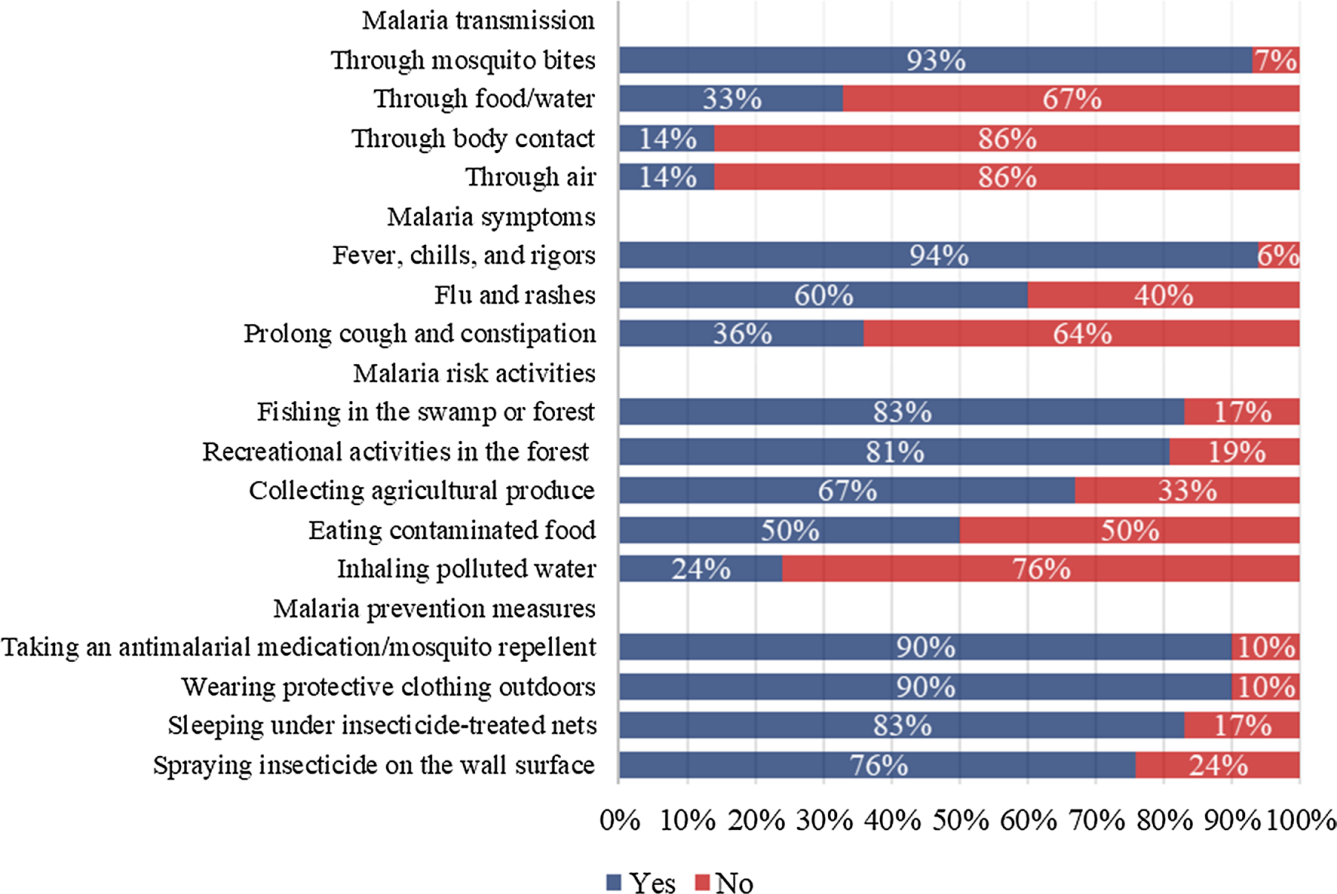
Response on malaria transmission, symptoms, risk activities, and prevention measures among respondents who had ever heard of malaria (n = 2491)

The assessment of knowledge of malaria transmission, symptoms, risk activities, and prevention measures was conducted on 2491 respondents who had ever heard of malaria (Fig. 1). The majority of respondents (93%) knew that malaria was transmitted through mosquito bites. However, some respondents believed that malaria could be transmitted through food/water (33%), body contact (14%), or air (14%). In addition, the majority of respondents (94%) identified fever, chills, and rigors as the main symptoms of malaria. About three-fifths of respondents reported flu and rashes (60%), and 36% reported prolonged cough and constipation as symptoms of malaria. Regarding the risk activities that lead to malaria, most respondents identified fishing in the swamp or forest (83%), recreational activities in the forest (81%), and collecting agricultural products (67%) as risk activities. However, there were also respondents who identified eating contaminated food (50%) and inhaling polluted water (24%) as risky activities that lead to malaria. Regarding malaria prevention measures, the majority of respondents indicated that taking an anti-malarial medication or using mosquito repellents during activities in the forest area (90%), wearing protective clothing during activities outdoors or in the farm/forest area (90%), sleeping under insecticide-treated nets (83%), and spraying insecticide on the wall surface (76%) as malaria prevention measures.

### Respondent’s correct knowledge of malaria

Table 2 shows the prevalence of correct knowledge of malaria transmission, symptoms, risk activities, and prevention measures among those who ever heard of malaria. The overall prevalence of ever heard of malaria in Malaysia was 76.1% (95% CI 70.5, 80.9). People living in urban areas showed a lower prevalence at 74.6% (95% CI 67.7, 80.4) than those living in rural areas at 81.3% (95% CI 75.8, 85,8). There was no significant difference between these two locations. Among those who had ever heard of malaria, the prevalence of correct knowledge of malaria transmission was 53.9% (95% CI 50.2, 57.7). It was higher in urban areas [55.2% (95% CI 50.5, 59.7)] than in rural areas [50.1% (95% CI 44.3, 55.9)]. As for the prevalence of correct knowledge of malaria symptoms, the overall prevalence was 24.1% (95% CI 21.2, 27.2). People living in rural areas have a higher prevalence at 26.5% (95% CI 23.3, 29.9) than those living in urban [23.3% (95% CI 19.7, 27.3)]. Subsequently, the overall prevalence of correct knowledge about malaria risky activities was 34.0% (95% CI 29.3, 39.1). People living in urban areas have a higher prevalence at 36.2% (95% CI: 30.3, 42.6) than those living in rural [27.1% (95% CI 22.2, 32.6)]. For the prevalence of correct knowledge about prevention measures, the overall prevalence was 59.7% (95% CI 55.5; 63.7). It was higher in rural areas [63.2% (95% CI 58.2, 67.9)] than in urban areas [58.5% (95% CI 53.3, 63.6)].

**Table 2:** Prevalence of correct knowledge of malaria transmission, symptoms, risk activities, and prevention measures among those who had ever heard of malaria

Item	Malaysia			Urban			Rural		
	n	N	Prevalence (95% CI)	n	N	Prevalence (95% CI)	n	N	Prevalence (95% CI)
Ever heard of Malaria	2491	18,803,830	76.1 (70.5–80.9)	1379	14,255,550	74.6 (67.7–80.4)	1112	4,548,280	81.3 (75.8–85.8)
Correct Transmission	1361	10,144,441	53.9 (50.2–57.7)	771	7,865,398	55.2 (50.5–59.7)	590	2,279,043	50.1 (44.3–55.9)
Correct Symptoms	658	4,527,257	24.1 (21.2–27.2)	350	3,323,253	23.3 (19.7–27.3)	308	1,204,104	26.5 (23.3–29.9)
Correct Risk activities	757	6,392,053	34.0 (29.3–39.1)	463	5,160,038	36.2 (30.3–42.6)	294	1,232,015	27.1 (22.2–32.6)
Correct prevention measures	1522	11,217,161	59.7 (55.5–63.7)	814	8,344,802	58.5 (53.3–63.6)	708	2,872,360	63.2 (58.2–67.9)

**Fig. 2:**
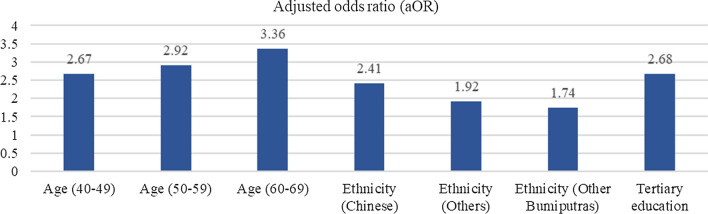
Significance factors of correct answer associated with malaria transmission

The association between the level of correct knowledge of malaria and socio-demographic characteristics was tested using chi-square bivariate analysis to determine the variables to be included in the logistic regression model (Fig. 2 to Fig. 5). The results from Fig. 2 showed that older age respondents exhibited higher odds of correct knowledge, with those who were 40–49 (aOR = 2.67), 50–59 (aOR = 2.92), and 60–69 (aOR = 3.36) years older being more likely to have correct knowledge of malaria transmission compared to those 15–19 years old. In terms of ethnicity, it was reported that Chinese (aOR = 2.41) had higher odds of correct knowledge of malaria transmission, followed by other ethnicities (aOR = 1.92) and other Bumiputras (aOR = 1.74) as compared to the Indians. In addition, respondents with tertiary educational levels were 2.68 times more likely to have correct knowledge of malaria transmission than people who had no formal education.

**Fig. 3:**
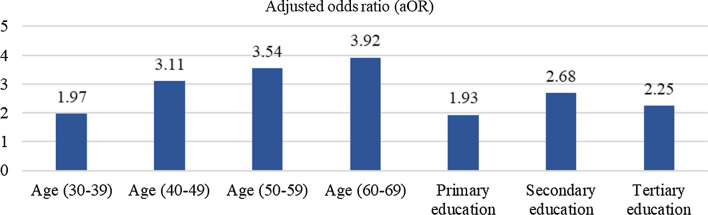
Significance factors of correct answer associated with malaria symptoms

**Fig. 4:**
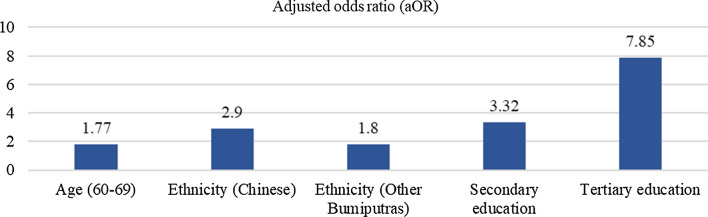
Significance factors of correct answer associated with malaria risk activities

**Fig. 5:**
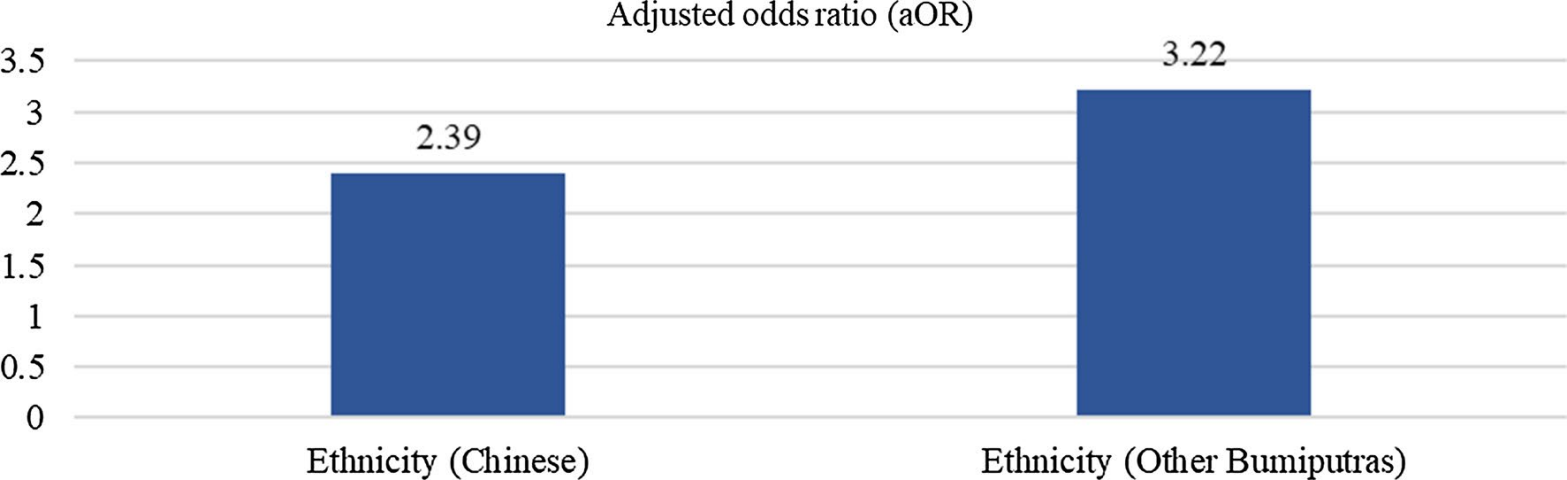
Significance factors of correct answer associated with malaria prevention measures

Figure 3 shows that the older the person, the higher the odds of having correct knowledge of malaria symptoms; 30–39 (aOR = 1.97), 40–49 (aOR = 3.11), 50–59 (aOR = 3.54), and 60–69 (aOR = 3.92) years old compared to those aged 15–19 years old. The odds of having correct knowledge of malaria symptoms were higher among those with higher education status; those who had obtained at least primary education (aOR = 1.93), secondary education (aOR = 2.68) or tertiary education (aOR = 2.25) were more likely to have correct knowledge of malaria symptoms than those without formal education. Figure 4 shows that having correct knowledge of malaria risk activities was significantly higher among those aged 60—69 years (aOR = 1.77). In terms of ethnicity, the Chinese (aOR = 2.90) and other Bumiputras (aOR = 1.80) were more likely to have correct knowledge of malaria risk activities than the Indians. The odds of having correct knowledge of malaria risk activities increased as the educational level increased; those with secondary (aOR = 3.32) and tertiary (aOR = 7.85) education are more likely to have correct knowledge of malaria risk activities compared to those without formal education. The results from Fig. 5 showed that Chinese and other Bumiputras had 2.39 and 3.22 times, respectively correct knowledge of malaria prevention measures compared to the Indians.

## Discussion

More than two-thirds of Malaysians had ever heard of malaria disease. This prevalence was lower than in other malaria-eliminating countries such as Cambodia and Nepal which reported a prevalence of 99% and 86% respectively [18, 19]. However, both surveys were conducted in malaria-endemic areas, where information pertaining to malaria knowledge and awareness are pertinent. In contrast, this survey was conducted among the Malaysian general population where the low and no incidence of malaria areas were also included. This is because, in order to sustain malaria elimination programmes in the country, broad coverage of malaria knowledge is required to prevent infection from risk activities associated with malaria. Findings from a survey of malaria low-incidence areas in rural India found that all (100%) of its respondents knew about malaria, and this implies that having adequate knowledge of malaria could sustain malaria prevention in the community [20].

Although this survey found that 93% of respondents believed that malaria transmission was caused by mosquito bites, the prevalence of correct knowledge regarding malaria transmission was quite low (54%), with a small proportion still believing that malaria could be transmitted through food/water (33%), body contact (14%), and air (14%). This is consistent with previous surveys which reported that most respondents were aware of the role of mosquitoes in transmitting malaria and will be more vigilant to avoid mosquito bites [21–23]. However, other surveys reported that knowledge of malaria transmission among respondents was low, even though the study was conducted in malaria-endemic areas [24–26]. Therefore, when planning a malaria control strategy, understanding the characteristics of malaria vector mosquitoes such as biting time, feeding behaviour, seasonal abundance patterns, breeding, and resting sites, including the influence of meteorological parameters, should be considered so that the results are efficient and effective. An appropriate planning plan for routine vector control should be implemented so that activities are performed at the time when vectors are actively biting. These results are important for malaria management programmes to facilitate the design of malaria vector control programmes that are relevant to local characteristics, including the vectors and their activity patterns [27].

The majority of respondents (94%) were able to identify the most common malaria symptoms (fever, chills, and rigors), but correct knowledge of malaria symptoms in the population was estimated to be very low (24%). The symptoms such as flu, rashes, prolonged cough, and constipation have been misinterpreted as malaria symptoms. However, the findings were considered high compared to previous surveys conducted in malaria-endemic areas in Peninsular Malaysia [12, 13]. This is important as a good knowledge of malaria symptoms will encourage communities to seek immediate treatment to avoid serious complications [22, 28].

Regarding the knowledge of risk activities leading to malaria transmission, this survey showed that most respondents knew about malaria risk activities such as fishing in the swamp or forest, recreational activities in the forest, and collecting agricultural produce. However, correct knowledge of malaria risk activities was rated as low (34%), as misconceptions about malaria risk activities such as eating contaminated food and inhaling polluted water were still reported. This is similar to the results of a survey in Bangladesh, which found that respondents opined their risk activities did not expose them to malaria transmission [29]. In terms of knowledge of malaria prevention measures, most respondents knew what prevention measures could be taken to prevent malaria transmission. More than half of the respondents had a good knowledge of malaria prevention measures, which was the highest level among other knowledge. A previous survey showed that although respondents had a higher level of knowledge about prevention measures, a significant number of them (43%) did not take personal protective measures to protect against malaria infection [22]. This issue needs to be addressed to raise awareness of malaria eradication among the community, particularly in malaria-endemic areas.

This survey revealed that rural communities exhibit a higher level of malaria awareness and the correct knowledge regarding symptoms and prevention measures. Rural communities, particularly those residing in malaria-endemic areas, frequently display a heightened level of awareness concerning malaria due to direct exposure and personal encounters with the disease. Consequently, they often possess firsthand experiences or exposures to malaria cases, leading to enhanced awareness and comprehension of the disease, as well as appropriate preventive actions [20, 24]. Conversely, urban communities demonstrate a better understanding of malaria transmission and the risky activities associated with malaria infection. This is commonly attributed to their increased exposure to health education campaigns, the dissemination of information, and enhanced access to healthcare facilities. Urban populations may have access to public health messages, media campaigns, and educational materials that emphasize the significance of avoiding mosquito bites, utilizing bed nets, and undertaking preventive measures [21, 30]. Furthermore, urban areas may benefit from more structured and organized public health interventions that target specific populations or areas at a higher risk of malaria transmission [31].

This survey found that age, ethnicity, and educational level were significantly associated with correct malaria transmission and risk activities. In addition, age and educational level were significantly associated with correct malaria symptoms. Meanwhile, only the variable ethnicity showed a significant association with the correct malaria prevention measures. Older people are expected to be more exposed to malaria knowledge than young people as malaria control programs have been put in place and given more emphasis in the past [3]. Younger generations were not exposed to malaria as many countries had almost eliminated malaria. These findings were consistent with several surveys conducted among indigenous peoples in Malaysia [12, 14]. In addition, the older age group had more experience with the disease and participated more frequently in awareness-raising activities [32]. On the other hand, previous surveys in Ethiopia [26], Thailand [33], and Nigeria [34] showed that young people have better knowledge of malaria than older people. This is because sources of information are now easily accessible to the younger generation through schools or mass media such as television and the Internet. In addition, the educational attainment of the younger generation is higher in most countries due to increased awareness of the importance of education [33]. Sustaining public awareness of malaria requires a comprehensive strategy, prominently emphasizing continuous educational and awareness campaigns. These initiatives encompass critical information on disease characteristics, transmission modes, prevention strategies, and the critical significance of early detection and treatment. Moreover, the effective integration of malaria education into school curricula and community programs can be achieved through active participation in a diverse range of interactive activities, workshops, and seminars [35]. This proactive approach ensures that younger generations develop a thorough understanding of the risks associated with the disease, thereby fortifying their capacity to counteract its potential resurgence.

Educated people are discussed as a possible explanation for better knowledge of malaria than the uneducated [36]. Previous surveys have shown that knowledge of malaria was slightly better in areas with higher levels of education among respondents [34, 37]. In fact, educated people are more likely to be reached by malaria messages on various audio-visual platforms such as television, radio, newspaper, internet, while uneducated people are not [38]. A survey in Nigeria found that uneducated people are more likely to seek treatment from unauthorized practitioners or buy medications from street vendors [34]. The association of knowledge of malaria transmission with ethnicity was justified when certain ethnic groups living in malaria-endemic areas had better knowledge of the mode of malaria transmission [39]. In addition, people living in malaria-endemic areas had a higher knowledge of malaria compared to people living in non-endemic areas [33].

Nevertheless, this survey might have some limitations that need to be highlighted. The survey was conducted during the peak of the COVID-19 pandemic in 2020. Therefore, the interviews were conducted via computer-assisted telephone interviews (CATI). Even though CATI was used, the interviewers were trained to ensure interviews were done according to the study protocol, which could limit information bias from the survey. This survey also included selected respondents from the general population and did not focus on a specific group of population living in the malaria-endemic areas. Even though the malaria prevention programme is important for people who live in areas where malaria is common, this information could be used to spread policies and programmes for reducing malaria across the country to make sure that malaria is eliminated from the country. This survey also provides a large sample size for analysis, so the analysis can describe the weighted estimates for all Malaysian states.

## Conclusion

In general, the prevalence of ever heard of malaria was considered high since the survey was performed based on a non-specific malaria population. However, the prevalence of correct knowledge of malaria symptoms and risk activities was considered inadequate. It is concluded that educational level is one of the most important factors for correct knowledge of malaria, along with age, ethnicity, and occupation. Therefore, targeted research or educational innovation targeting specific vulnerable communities or groups should be the way forward. This survey successfully identified the specific age, ethnicity, educational level, and occupation that malaria programme managers could focus on in preparing an initiative for sustainable malaria elimination.

### Supplementary Information

**Additional file 1: Table S1** The distribution of the valid survey respondents in each state of Malaysia. **Table S2 **Estimates of crude and adjusted odds ratios for the factors of correct answer associated with malaria transmission.

## Data Availability

The dataset used and/or analysed during the current survey are available from the corresponding authors upon request. Availability of data can be requested by contacting faizul.abdrahim@moh.gov.my.

## Abbreviations

NHMS: National Health and Morbidity Survey
CATI: Computer-assisted telephone interviews
WHO: World Health Organization
MOH: Ministry of Health

## References

[1] WHO (2020). World malaria report 2020: 20 years of global progress and challenges.

[2] WHO (2015). Global technical strategy for malaria 2016–2030.

[3] Mudin RN. Malaria: battling old disease with new strategies. Presented during 5^th^ Perak Health Conference. Ipoh, Perak, Malaysia. 2013.

[4] William T, Menon J (2014). A review of malaria research in Malaysia. Med J Malaysia.

[5] Nchinda TC (1998). Malaria: a reemerging disease in Africa. Emerg Infect Dis.

[6] Govere J, Durrheim D, La Grange K, Mabuza A, Booman M (2000). Community knowledge and perceptions about malaria and practices influencing malaria control in Mpumalanga Province. South Africa South African Med J.

[7] Kroeger A, Meyer R, Mancheno M, Gonzalez M, Pesse K (1997). Operational aspects of bednet impregnation for community-based malaria control in Nicaragua, Ecuador, Peru and Colombia. Trop Med Int Health.

[8] Klein RE, Weller SC, Zeissig R, Richards FO, Ruebush TK (1995). Knowledge, beliefs, and practices in relation to malaria transmission and vector control in Guatemala. Am J Trop Med Hyg.

[9] Keating J, Eisele TP, Bennett A, Johnson D, Macintyre K (2008). A description of malaria-related knowledge, perceptions, and practices in the Artibonite Valley of Haiti: implications for malaria control. Am J Trop Med Hyg.

[10] Uganda Bureau of Statistics, ICF Macro (Firm). Uganda Malaria Indicator Survey, 2009. Uganda Bureau of Statistics; 2010. https://dhsprogram.com/pubs/pdf/MIS6/MIS6.pdf.

[11] Keiser J, Utzinger J, Caldas de Castro M, Smith TA, Tanner M, Singer BH (2004). Urbanization in sub-saharan Africa and implication for malaria control. Am J Trop Med Hyg.

[12] Al-Adhroey AH, Nor ZM, Al-Mekhlafi HM, Mahmud R (2010). Opportunities and obstacles to the elimination of malaria from Peninsular Malaysia: knowledge, attitudes and practices on malaria among aboriginal and rural communities. Malar J.

[13] Munajat MB, Rahim MA, Wahid W, Seri Rakna MI, Divis P, Chuangchaiya S (2021). Perceptions and prevention practices on malaria among the indigenous Orang Asli community in Kelantan. Peninsular Malaysia Malar J.

[14] Kader Maideen SF, Rashid A, Ahmad NI, Zahari SN, Hamat RA (2022). Sero-prevalence of malaria and the knowledge, attitudes and practices relating to the prevention of malaria among indigenous people living in the central forest spine in Peninsular Malaysia: a mixed-methods study. Malar J.

[15] Institute for Public Health. 2021. National Health and Morbidity Survey (NHMS) 2020: Communicable Diseases. Vol. 1. 280 pages. Shah Alam, Selangor, Malaysia.

[16] Soleimani-Ahmadi M, Vatandoost H, Zare M, Alizadeh A, Salehi M (2014). Community knowledge and practices regarding malaria and long-lasting insecticidal nets during malaria elimination programme in an endemic area in Iran. Malar J.

[17] WHO (2005). Malaria indicator survey: basic documentation for survey design and implementation/Roll Back Malaria Monitoring and Evaluation Reference Group.

[18] Kheang ST, Por I, Sovannaroth S, Dysoley L, Chea H, Po L (2021). Cambodia malaria indicator survey 2020: implications for malaria elimination. MalarWorld J.

[19] Joshi AB, Banjara MR (2008). Malaria related knowledge, practices and behaviour of people in Nepal. J Vector Borne Dis.

[20] Gupta RK, Raina SK, Shora TN, Jan R, Sharma R, Hussain S (2016). A household survey to assess community knowledge, attitude and practices on malaria in a rural population of Northern India. J Family Med Prim Care.

[21] Yaya S, Bishwajit G, Ekholuenetale M, Shah V, Kadio B, Udenigwe O (2017). Knowledge of prevention, cause, symptom and practices of malaria among women in Burkina Faso. PLoS ONE.

[22] Hlongwana KW, Mabaso ML, Kunene S, Govender D, Maharaj R (2009). Community knowledge, attitudes and practices (KAP) on malaria in Swaziland: a country earmarked for malaria elimination. Malar J.

[23] Bamaga OA, Mahdy MA, Mahmud R, Lim YA (2014). Malaria in Hadhramout, a southeast province of Yemen: prevalence, risk factors, knowledge, attitude and practices (KAPs). Parasit Vectors.

[24] Mazigo HD, Obasy E, Mauka W, Manyiri P, Zinga M, Kweka EJ (2010). Knowledge, attitudes, and practices about malaria and its control in rural northwest Tanzania. Malar Res Treat.

[25] Sanjana P, Barcus MJ, Bangs MJ, Ompusunggu S, Elyazar I, Marwoto H (2006). Survey of community knowledge, attitudes, and practices during a malaria epidemic in central Java. Indonesia Am J Trop Med Hyg.

[26] Paulander J, Olsson H, Lemma H, Getachew A, San SM (2009). Knowledge, attitudes and practice about malaria in rural Tigray. Ethiopia Glob Health Action.

[27] Rahim FA, Mutalip MH, Hasim MH, Mahmud MA, Yeop N (2019). Factors associated with distribution of *Anopheles sundaicus* in coastal area, Kuala Penyu. Sabah Int J Academ Res Develop.

[28] Faye O, Lo M, Diop B, Gaye O, Bah I, Dieng T, Dieng Y, N’Dir O, Diallo S (1997). Knowledge and treatment of malaria in rural Senegal (in French). Med Trop.

[29] Saha A, Sarker M, Kabir M, Lu G, Müller O (2019). Knowledge, attitudes, and practices regarding malaria control among the slash and burn cultivators in Rangamati Hill tracts of Bangladesh. Malar J.

[30] Oguonu T, Okafor HU, Obu HA (2005). Caregivers’s knowledge, attitude and practice on childhood malaria and treatment in urban and rural communities in Enugu, south-east Nigeria. Public Health.

[31] Romay-Barja M, Jarrin I, Ncogo P, Nseng G, Sagrado MJ, Santana-Morales MA (2015). Rural-urban differences in household treatment-seeking behaviour for suspected malaria in children at Bata District. Equatorial Guinea PLoS One.

[32] Aung PL, Pumpaibool T, Soe TN, Kyaw MP (2019). Knowledge, attitude and practice levels regarding malaria among people living in the malaria endemic area of Myanmar. J Health Res.

[33] van Benthem BH, Khantikul N, Panart K, Somboon P, Oskam L (2006). Knowledge and use of preventive measures against malaria in endemic and non-endemic villages in northern Thailand. Southeast Asian J Trop Med Public Health.

[34] Dawaki S, Al-Mekhlafi HM, Ithoi I, Ibrahim J, Atroosh WM, Abdulsalam AM (2016). Is Nigeria winning the battle against malaria? Prevalence, risk factors and KAP assessment among Hausa communities in Kano State. Malar J.

[35] Beier JC, Keating J, Githure JI, Macdonald MB, Impoinvil DE, Novak RJ (2008). Integrated vector management for malaria control. Malar J.

[36] Yeneneh H, Gyorkos TW, Joseph L, Pickering J, Tedla S (1993). Antimalarial drug utilization by women in Ethiopia: a knowledge, attitudes, practice study. Bull World Health Organ.

[37] Rodriguez AD, Penilla RP, Henry-Rodriguez M, Hemingway J, Francisco Betanzos A, Hernandez-Avila JE (2003). Knowledge and beliefs about malaria transmission and practices for vector control in southern Mexico. Salud Publica Mex.

[38] Kala Chouakeu NA, Ngingahi LG, Bamou R, Talipouo A, Ngadjeu CS, Mayi MP (2021). Knowledge, attitude, and practices (KAP) of human populations towards malaria control in four ecoepidemiological settings in Cameroon. J Trop Med.

[39] Canavati SE, Kelly GC, Quintero CE, Vo TH, Tran LK, Ohrt C (2019). Risk factor assessment for clinical malaria among forest-goers in a pre-elimination setting in Phu Yen Province. Vietnam Malar J.

