# Supplementary material for: Assessing the population’s correct knowledge of malaria in Malaysia: a vital component for malaria elimination certification
Source: Malar J. 2023 Sep 12;22:267. doi: 10.1186/s12936-023-04704-1 (PMC10496336; doi:10.1186/s12936-023-04704-1)
Supplement: Supplementary file 1 — Additional file 1: Table S1 The distribution of the valid survey respondents in each state of Malaysia. Table S2 Estimates of crude and adjusted odds ratios for the factors of correct answer associated with malaria transmission. [file 12936_2023_4704_MOESM1_ESM.doc]

**Table 1** The distribution of the valid survey respondents in each state of Malaysia

| **No** | **State** | **No. of respondent** | | |
| --- | --- | --- | --- | --- |
|  |  | **Urban** | **Rural** | **Total** |
| 1 | Johor | 141 | 175 | 316 |
| 2 | Kedah | 124 | 107 | 231 |
| 3 | Kelantan | 86 | 142 | 228 |
| 4 | Melaka | 93 | 52 | 145 |
| 5 | Negeri 9 | 111 | 102 | 213 |
| 6 | Pahang | 46 | 51 | 97 |
| 7 | Penang | 45 | 44 | 89 |
| 8 | Perak | 88 | 61 | 149 |
| 9 | Perlis | 38 | 29 | 67 |
| 10 | Selangor | 284 | 110 | 394 |
| 11 | Terengganu | 38 | 46 | 84 |
| 12 | Sabah | 189 | 202 | 391 |
| 13 | Sarawak | 226 | 246 | 472 |
| 14 | KL | 147 | 0 | 147 |
| 15 | Labuan | 41 | 0 | 41 |
| 16 | Putrajaya | 21 | 0 | 21 |
|  |  | 1718 | 1367 | 3085 |

**Table 2** Estimates of crude and adjusted odds ratios for the factors of correct answer associated with malaria transmission

| **Variable** | **Crude OR (95% CI)** | **P-value** | **Adjusted OR (95%CI)** | **P-value** |
| --- | --- | --- | --- | --- |
| **Sex** |  |  |  |  |
| Male | 1.06 (0.82-1.36) | 0.662 |  |  |
| Female (ref) | 1 |  |  |  |
| **Strata** |  |  |  |  |
| Urban | 1.23 (0.95-1.58 | 0.111 |  |  |
| Rural (ref) | 1 |  |  |  |
| **Age** |  |  |  |  |
| 15-19 (ref) | 1 |  | 1 |  |
| 20-29 | 1.80 (1.19-2.74) | 0.008* | 1.40 (0.91-2.17) | 0.125 |
| 30-39 | 2.66 (1.76-4.02) | <0.001* | 1.83 (0.97-3.43) | 0.06 |
| 40-49 | 3.57 (2.28-5.59) | <0.001* | 2.67 (1.05-6.78) | 0.039* |
| 50-59 | 3.63 (2.28-5.78) | <0.001* | 2.92 (1.06-8.09) | 0.04* |
| 60-69 | 3.97 (2.90-5.44) | <0.001* | 3.36 (1.18-9.61) | 0.025* |
| 70 & above | 2.45 (1.56-3.84) | <0.001* | 2.27 (0.81-6.36) | 0.115 |
| **Ethnicity** |  |  |  |  |
| Indian (ref) | 1 |  | 1 |  |
| Malay | 1.33 (0.96-1.84) | 0.084 | 1.30 (0.97-1.75) | 0.079 |
| Chinese | 2.60 (1.58-4.29) | 0.001* | 2.41 (1.50-3.88) | 0.001* |
| Other Bumiputras | 1.40 (0.97-2.04) | 0.072 | 1.74 (1.25-2.42) | 0.002* |
| Others | 1.18 (0.71-1.96) | 0.518 | 1.92 (1.15-3.20) | 0.008* |
| **Marital Status** |  |  |  |  |
| Single (ref) | 1 |  | 1 |  |
| Married/Living with a partner | 1.77 (1.35-2.33) | <0.001* | 1.15 (0.45-2.92) | 0.768 |
| Widowed (er)/divorcee | 2.12 (1.49-3.00) | <0.001* | 1.32 (0.49-3.56) | 0.574 |
| **Education Level** |  |  |  |  |
| No formal education (ref) | 1 |  | 1 |  |
| Primary education | 1.32 (0.82-2.14) | 0.247 | 1.34 (0.78-2.29) | 0.271 |
| Secondary education | 1.36 (0.87-2.12) | 0.168 | 1.59 (0.93-2.73) | 0.09 |
| Tertiary education | 2.33 (1.52-3.56) | <0.001* | 2.68 (1.57-4.56) | 0.001* |
| **Occupation** |  |  |  |  |
| Student (ref) | 1 |  | 1 |  |
| Government employee | 3.57 (2.36-5.42) | <0.001* | 1.52 (0.72-3.20) | 0.256 |
| Private employee | 1.86 (1.18-2.93) | 0.009* | 0.99 (0.58-1.70) | 0.975 |
| Self-employed | 1.87 (1.37-2.56) | <0.001* | 0.99 (0.66-1.49) | 0.984 |
| Unpaid worker/Homemaker/caregiver | 1.78 (1.18-2.69) | 0.008* | 0.93 (0.42-2.05) | 0.859 |
| Not working (unemployed, health problem, old age, retiree) | 2.21 (1.68-2.91) | <0.001* | 1.03 (0.72-1.46) | 0.872 |
| Others | 1.17 (0.23-6.01) | 0.842 | 0.83 (0.18-3.78) | 0.801 |
| Adjusted Wald Test for all parameters: F (21,7) =84.19 p<0.001. | | | | |
| goodness of fit=0.9881 | | | | |
| *Significance at P< 0.05 | | | | |

**Table 3** Estimates of crude and adjusted odds ratios for the factors of correct answer associated with malaria symptoms

| **Variable** | **Crude OR (95% CI)** | **P-value** | **Adjusted OR (95% CI)** | **P-value** |
| --- | --- | --- | --- | --- |
| **Sex** |  |  |  |  |
| Male | 1.00 (0.86-1.16) | 0.968 |  |  |
| Female (ref) | 1 |  |  |  |
| **Strata** |  |  |  |  |
| Urban | 0.84 (0.65-1.10) | 0.201 |  |  |
| Rural (ref) | 1 |  |  |  |
| **Age** |  |  |  |  |
| 15-19 (ref) | 1 |  | 1 |  |
| 20-29 | 0.97 (0.52-1.80) | 0.925 | 1.20 (0.70-2.05) | 0.502 |
| 30-39 | 1.82 (0.98-3.38) | 0.057 | 1.97 (1.08-3.60) | 0.029* |
| 40-49 | 2.93 (1.53-5.63) | 0.002* | 3.11 (1.25-7.73) | 0.017* |
| 50-59 | 3.26 (1.85-5.72) | <0.001* | 3.54 (1.57-7.99) | 0.004* |
| 60-69 | 3.71 (2.15-6.39) | <0.001* | 3.92 (1.67-9.21) | 0.003* |
| 70 & above | 2.41 (0.88-6.62) | 0.086 | 2.57 (0.72-9.15) | 0.139 |
| **Ethnicity** |  |  |  |  |
| Indian (ref) | 1 |  |  |  |
| Malay | 1.37 (0.82-2.30) | 0.216 |  |  |
| Chinese | 0.83 (0.42-1.67) | 0.598 |  |  |
| Other Bumiputras | 1.25 (0.77-2.02) | 0.358 |  |  |
| Others | 1.26 (0.50-3.16) | 0.608 |  |  |
| **Marital Status** |  |  |  |  |
| Single (ref) | 1 |  | 1 |  |
| Married/Living with a partner | 2.34 (1.77-3.11) | <0.001* | 1.51 (0.91-2.51) | 0.109 |
| Widowed (er)/divorcee | 3.59 (2.20-5.86) | <0.001* | 2.13 (0.99-4.57) | 0.052 |
| **Education Level** |  |  |  |  |
| No formal education (ref) | 1 |  | 1 |  |
| Primary education | 2.33 (1.07-5.06) | 0.034* | 1.93 (1.08-3.45) | 0.028* |
| Secondary education | 2.70 (0.98-7.40) | 0.053 | 2.68 (1.35-5.34) | 0.007* |
| Tertiary education | 2.06 (0.74-5.73) | 0.157 | 2.25 (1.08-4.68) | 0.032* |
| **Occupation** |  |  |  |  |
| Student (ref) | 1 |  | 1 |  |
| Government employee | 2.12 (1.23-3.65) | 0.009* | 0.66 (0.34-1.28) | 0.209 |
| Private employee | 1.43 (0.90-2.28) | 0.126 | 0.58 (0.35-0.98) | 0.044* |
| Self-employed | 1.56 (1.04-2.35) | 0.034 | 0.47 (0.23-0.99) | 0.046* |
| Unpaid worker/Homemaker/caregiver | 2.07 (1.39-3.06) | 0.001 | 0.61 (0.37-1.03) | 0.064 |
| Not working (unemployed, health problem, old age, retiree) | 2.17 (1.35-3.50) | 0.002 | 0.58 (0.26-1.27) | 0.162 |
| Others | 1.71 (0.24-12.32) | 0.58 | 1.11 (0.18-6.88) | 0.912 |
| Adjusted Wald Test for all parameters: F (18,10) =17.50 p<0.001. | | | | |
| goodness of fit=0.2928 | | | | |
| *Significance at P< 0.05 | | | | |

**Table 4** Estimates of crude and adjusted odds ratios for the factors of correct answer associated with malaria risk activities

| **Variable** | | **Crude OR (95% CI)** | **P-value** | | **Adjusted OR (95% CI)** | | **P-value** | |
| --- | --- | --- | --- | --- | --- | --- | --- | --- |
| **Sex** | |  |  | |  | |  | |
| Male | | 1.12 (0.89-1.39) | 0.318 | |  | |  | |
| Female (ref) | | 1 |  | |  | |  | |
| **Strata** | |  |  | |  | |  | |
| Urban | | 1.52 (1.05-2.23) | 0.029* | | 1.06 (0.81-1.40) | | 0.661 | |
| Rural (ref) | | 1 |  | | 1 | |  | |
| **Age** | |  |  | |  | |  | |
| 15-19 (ref) | | 1 |  | | 1 | |  | |
| 20-29 | | 1.20 (0.76-1.89) | 0.419 | | 0.98 (0.70-1.36) | | 0.891 | |
| 30-39 | | 1.57 (0.85-2.88) | 0.14 | | 1.28 (0.74-2.21) | | 0.359 | |
| 40-49 | | 1.47 (0.98-2.20) | 0.061 | | 1.38 (0.88-2.15) | | 0.15 | |
| 50-59 | | 1.44 (1.2-2.05) | 0.041* | | 1.64 (0.94-2.86) | | 0.081 | |
| 60-69 | | 1.34 (0.86-2.08) | 0.182 | | 1.77 (1.00-3.13) | | 0.049* | |
| 70 & above | | 0.62 (0.27-1.40) | 0.237 | | 0.99 (0.35-2.81) | | 0.992 | |
| **Ethnicity** | |  |  | |  | |  | |
| Indian (ref) | | 1 |  | | 1 | |  | |
| Malay | | 1.19 (0.62-2.31) | 0.585 | | 1.03 (0.59-1.80) | | 0.905 | |
| Chinese | | 3.41 (1.55-7.49) | 0.004* | | 2.90 (1.37-6.12) | | 0.007* | |
| Other Bumiputras | | 1.47 (0.77-2.82) | 0.23 | | 1.80 (1.04-3.12) | | 0.037* | |
| Others | | 0.59 (0.21-1.63) | 0.297 | | 0.92 (0.40-2.15) | | 0.849 | |
| **Marital Status** | |  |  | |  | |  | |
| Single (ref) | | 1 |  | |  | |  | |
| Married/Living with a partner | | 1.06 (0.85-1.33) | 0.6 | |  | |  | |
| Widowed (er)/divorcee | | 0.89 (0.61-1.30) | 0.543 | |  | |  | |
| **Education Level** | |  |  | |  | |  | |
| No formal education (ref) | | 1 |  | | 1 | |  | |
| Primary education | | 2.26 (0.68-7.53) | 0.176 | | 1.75 (0.63-4.88) | | 0.272 | |
| Secondary education | | 4.16 (1.15-15.03) | 0.031* | | 3.32 (1.12-9.87) | | 0.032* | |
| Tertiary education | | 9.99 (3.02-33.10) | 0.001* | | 7.85 (2.89-21.33) | | <0.001* | |
| **Occupation** | |  |  | |  | |  | |
| Student (ref) | | 1 |  | | 1 | |  | |
| Government employee | | 1.30 (0.80-2.14) | 0.279 | | 0.72 (0.37-1.41) | | 0.324 | |
| Private employee | | 1.04 (0.59-1.85) | 0.891 | | 0.78 (0.46-1.33) | | 0.35 | |
| Self-employed | | 0.77 (0.45-1.32) | 0.329 | | 0.66 (0.38-1.16) | | 0.144 | |
| Unpaid worker/Homemaker/caregiver | | 0.60 (0.38-0.95) | 0.031* | | 0.55 (0.30-1.03) | | 0.06 | |
| Not working (unemployed, health problem, old age, retiree) | | 0.73 (0.46-1.14) | 0.159 | | 0.58 (0.30-1.14) | | 0.111 | |
| Others | | 1.20 (0.26-5.63) | 0.808 | | 0.99 (0.22-4.59) | | 0.995 | |
| Adjusted Wald Test for all parameters: F (20,8) =77.29 p<0.001. | | | | | | | | |
| goodness of fit=0.5381 | | | | | | | | |
| *Significance at P< 0.05  **Table 5** Estimates of crude and adjusted odds ratios for the factors of correct answer associated with malaria prevention measures | | | | | | | | |
|  | **Variable** | **Crude OR (95% CI)** | **P-value** | **Adjusted OR (95% CI)** | | **P-value** | |  |
|  | **Sex** |  |  |  | |  | |  |
|  | Male | 0.93 (0.71-1.21) | 0.564 |  | |  | |  |
|  | Female (ref) | 1 |  |  | |  | |  |
|  | **Strata** |  |  |  | |  | |  |
|  | Urban | 0.82 (0.62-1.10) | 0.174 |  | |  | |  |
|  | Rural (ref) | 1 |  |  | |  | |  |
|  | **Age** |  |  |  | |  | |  |
|  | 15-19 (ref) | 1 |  |  | |  | |  |
|  | 20-29 | 0.86 (0.58-1.29) | 0.462 |  | |  | |  |
|  | 30-39 | 0.98 (0.73-1.32) | 0.89 |  | |  | |  |
|  | 40-49 | 0.73 (0.42-1.28) | 0.266 |  | |  | |  |
|  | 50-59 | 0.85 (0.52-1.37) | 0.49 |  | |  | |  |
|  | 60-69 | 0.84 (0.58-1.22) | 0.347 |  | |  | |  |
|  | 70 & above | 0.60 (0.33-1.08) | 0.084 |  | |  | |  |
|  | **Ethnicity** |  |  |  | |  | |  |
|  | Indian (ref) | 1 |  | 1 | |  | |  |
|  | Malay | 2.15 (0.95-4.87) | 0.065 | 2.11 (0.90-4.95) | | 0.085 | |  |
|  | Chinese | 2.39 (1.02-5.59) | 0.045* | 2.39 (1.00-5.66) | | 0.049* | |  |
|  | Other Bumiputras | 3.41 (1.55-7.48) | 0.003* | 3.22 (1.41-7.35) | | 0.007* | |  |
|  | Others | 1.52 (0.59-3.86) | 0.59-3.86 | 1.46 (0.53-4.04) | | 0.448 | |  |
|  | **Marital Status** |  |  |  | |  | |  |
|  | Single (ref) | 1 |  |  | |  | |  |
|  | Married/Living with a partner | 0.83 (0.68-1.02) | 0.08 |  | |  | |  |
|  | Widowed (er)/divorcee | 0.89 (0.56-1.41) | 0.608 |  | |  | |  |
|  | **Education Level** |  |  |  | |  | |  |
|  | No formal education (ref) | 1 |  |  | |  | |  |
|  | Primary education | 1.21 (0.69-2.10) | 0.49 |  | |  | |  |
|  | Secondary education | 1.30 (0.70-2.41) | 0.392 |  | |  | |  |
|  | Tertiary education | 1.37 (0.68-2.76) | 0.363 |  | |  | |  |
|  | **Occupation** |  |  |  | |  | |  |
|  | Student (ref) | 1 |  |  | |  | |  |
|  | Government employee | 1.06 (0.63-1.80) | 0.807 |  | |  | |  |
|  | Private employee | 0.76 (0.52-1.12) | 0.155 |  | |  | |  |
|  | Self-employed | 0.69 (0.44-1.08) | 0.1 |  | |  | |  |
|  | Unpaid worker/Homemaker/caregiver | 0.77 (0.56-1.05) | 0.099 |  | |  | |  |
|  | Not working (unemployed, health problem, old age, retiree) | 0.66 (0.42-1.04) | 0.07 |  | |  | |  |
|  | Others | 0.35 (0.07-1.63) | 0.172 |  | |  | |  |
|  | Adjusted Wald Test for all parameters: F (7,21) =6.12 p=0.0005 | | | | | | |  |
|  | goodness of fit=0.6077 | | | | | | |  |
|  | *Significance at P< 0.05 | | | | | | |  |
